# Developments in the management of psychosis: key evidence from 2022

**DOI:** 10.1192/j.eurpsy.2023.38

**Published:** 2023-07-19

**Authors:** N. Chakraborty

**Affiliations:** ^1^ Early intervention in psychosis, Leicestershire Partnership NHS Trust; ^2^Dept of Health Sciences, University of Leicester, Leicester, United Kingdom

## Abstract

**Abstract:**

The field of treatment and understanding in psychosis is evolving at an unprecedented pace with advances in neuroscience. It takes time before research findings are translated to clinical practice. Daunting as it is, clinicians need to keep up with findings which change our practice straightaway (new medication), findings which suggest that changes are expected in future (new theories about how illness develops and can be predicted) and findings which remind us of continuing practice and the evidence behind them (long term follow-up studies which give no big surprises but consolidate existing knowledge).

In a snap shot, I bring to you a range of five papers published in 2022 which enrich our understanding of psychosis, its development and treatment.

**Disclosure of Interest:**

None Declared

